# Infectious Disease Modeling and Military Readiness

**DOI:** 10.3201/eid1509.090702

**Published:** 2009-09

**Authors:** Brian H. Feighner, Stephen Eubank, Robert J. Glass, Victoria J. Davey, Jean-Paul Chrétien, Joel C. Gaydos

**Affiliations:** The Johns Hopkins University Applied Physics Laboratory, Laurel, Maryland, USA (B.H. Feighner); Virginia Polytechnic Institute and State University, Blacksburg, Virginia, USA (S. Eubank); Sandia National Laboratories, Albuquerque, New Mexico, USA (R. J. Glass); US Department of Veterans Affairs, Washington, DC, USA (V.J. Davey); Uniformed Services University of the Health Sciences, Bethesda, Maryland, USA (V.J. Davey); Walter Reed Army Institute of Research, Silver Spring, Maryland, USA (J.-P. Chrétien); US Department of Defense Armed Forces Health Surveillance Center, Silver Spring (J. C. Gaydos)

**Keywords:** Infectious disease, pandemic, influenza, modeling, military, conference summary

Advances in infectious disease modeling may offer opportunities to mitigate the effect of emerging infectious diseases upon military readiness (*1*–*3*). In August 2005, the US Department of Defense (DoD) Global Emerging Infections Surveillance and Response System (GEIS) sponsored a meeting on the epidemiologic applications of infectious disease modeling in support of DoD readiness. Several recommendations were made at this conference to include the identification of organizations with “…demonstrated expertise in model development and operation for collaboration with the DoD and civilian organizations that are developing simulation models or conducting exercises” (*4*). Despite this recommendation, infectious disease modeling efforts in support of DoD have remained somewhat disjointed. An infectious disease modeling collaboration between DoD-GEIS and The Johns Hopkins Applied Physics Laboratory, begun in 2007, again identified this issue. Concerned that opportunities for collaboration might be missed and that unintended redundancy might be occurring, DoD-GEIS sponsored a second conference on May 12–13, 2008, for infectious disease modelers engaged in DoD projects or on models useful to the DoD.

Over 30 participants from 10 agencies met for a day and a half at the Infectious Disease Modeling Meeting on the campus of the Johns Hopkins University Applied Physics Laboratory, Laurel, Maryland, USA (Appendix). The first day consisted of presentations detailing past and current work by the participating organizations. These presentations are available on the secure DoD-GEIS website for governmental organizations, collaborators, and academic institutions (request access from http://www.AFHSC.mil/about_GEIS.asp). The second day consisted of a roundtable discussion of how to optimize DoD-relevant infectious disease modeling efforts; specifically, how to maximize opportunities for collaboration and coordination while minimizing unintended redundancy.

The roundtable discussion first turned to who was at the table, and importantly, who was not. Many participants had also attended the 2005 conference, although some had not, and some of the key attendees at the 2005 conference were not present for the 2008 meeting. A strong recommendation was again made to identify all key organizations involved in infectious disease modeling of use to DoD. Additionally, because of the blurred lines of responsibility among federal agencies, participants thought that many other non-DoD federal organizations should participate in these types of discussions. Some participants called for the creation of a formal organization or society of infectious disease modelers. The Models of Infectious Disease Agent Study (MIDAS; http://www.nigms.nih.gov/Initiatives/MIDAS/Background/Factsheet.htm) group was mentioned as already serving as a nexus for the modeling community, but the need for a larger, coordinating body was expressed. Representatives from the Armed Forces Health Surveillance Center (the parent organization of DoD-GEIS; http://afhsc.army.mil/About_AFHSC.asp) indicated a willingness to be involved in future efforts in a coordinating capacity.

Participants also addressed the ambiguity associated with the term *modeling*, even within the infectious disease modeling community. Models may be used to provide indicators or warnings, surveillance data, or casualty prediction or to assist with consequence management, resource allocation, or policy development. Many models provide various combinations of these functions. Some participants believed it was important to organize modeling efforts by the functionality of the models in question. A stimulating discussion centered on who should have access to infectious disease models. Some thought that models should remain only in the hands of the experts who create them and can manage and interpret them. Others believed that state and community officials, who presumably have much greater local knowledge but less mathematical acumen, should be allowed access to the models.

The conference ended by reiterating the recommendation that the entire community of those working on disease modeling of interest to DoD should not only be identified but also strongly encouraged to meet again within the year. In addition to sharing ideas and work, participants of the 2008 conference recommended the development of a format and plan for ongoing communication and collaboration. This plan could include the formation of productive work groups to address definitions, e.g., the meaning of modeling, and to develop recommendations on the use of infectious disease models. The creation of a professional society for federal disease modelers could facilitate these actions and was identified for serious consideration.

## Appendix

Affiliations of conference participants at the Infectious Disease Modeling Meeting held May 12–13, 2008, at the Johns Hopkins University Applied Physics Laboratory:

Armed Forces Health Surveillance Center, US Department of Defense, Silver Spring, Maryland, USA
Defense Threat Reduction Agency, US Department of Defense, Alexandria, Virginia, USA
Global Emerging Infections Surveillance and Response System, US Department of Defense, Silver Spring, Maryland, USA
Johns Hopkins University Applied Physics Laboratory, Laurel, Maryland, USA
Quantum Leap Innovations, Inc., Arlington, Virginia, USA
Sandia National Laboratories, Albuquerque, New Mexico, USA
Uniformed Services University of the Health Sciences, Bethesda, Maryland, USA
US Department of Health and Human Services, Washington, DC, USA
US Department of Veterans Affairs, Washington, DC, USA
Virginia Bioinformatics Institute, Virginia Polytechnic Institute and State University, Blacksburg, Virginia, USA
